# Real-world burden of disease, treatment, and healthcare resource utilization in acromegaly: a quantitative survey of patient experiences

**DOI:** 10.1186/s41687-025-00965-3

**Published:** 2025-12-05

**Authors:** Maxwell Koobatian, Jill Sisco, Janetricks C. Okeyo, Alan Krasner, Tiffany P. Quock

**Affiliations:** 1 Crinetics Pharmaceuticals, Inc, San Diego, CA USA; 2 Acromegaly Community, Grove, OK USA

**Keywords:** Acromegaly* / drug therapy, Oral administration, Patient-reported outcomes, Somatostatin / analogs & derivatives, Symptom evaluation

## Abstract

**Background:**

Acromegaly is a rare endocrine disease caused by excessive growth hormone (GH) secretion typically due to a pituitary adenoma. Patients ineligible for or with an inadequate response to surgery and/or radiotherapy often require pharmacotherapy such as depot somatostatin receptor ligand (SRL) injections. This study evaluated the current management of patients with acromegaly and real-world experience of disease burden, treatment burden, and healthcare resource utilization (HCRU).

**Methodology:**

A quantitative study was conducted among symptomatic adults with acromegaly receiving therapy, including depot SRL injections. A web-based survey captured the 3-month disease experience including the presence and severity of acromegaly-associated symptoms, treatment experience, HCRU, and impact on the ability to work (Work Productivity and Impairment Questionnaire [WPAI], assessed over prior 7 days).

**Results:**

Among 58 patients who completed the survey, 36 (62.1%) received a depot SRL injection, either as monotherapy (18 [50%]), or in combination with other agents. All patients experienced ≥ 1 symptom during the previous 3 months, with 67% having ≥ 1 symptom with a severity of ≥ 8 on a scale of 0 to 10. Patients reported that acromegaly had a moderate (59%) or high (22%) level of interference in their life overall. Of 18 patients on depot SRL injection monotherapy, 12 (67%) reported ≥ 1 breakthrough acromegaly symptom at any time prior to the next injection. WPAI scores were 51% for daily activity impairment, 38% for presenteeism, 34% for overall work impairment, and 8% for absenteeism. Patients receiving depot SRL injection (monotherapy/combination therapy) were more likely than those not receiving this injection to report moderate-to-high interference of acromegaly treatment with their life (56%/72% vs. 18%) and that their treatment was moderately to highly burdensome (67%/72% vs. 41%). HCRU due to acromegaly treatment included: a mean of 2.6 office visits, 13.8% with ≥ 1 emergency department visit, and 10.3% with ≥ 1 overnight hospitalization. When asked about treatment preferences, 60% of patients preferred oral therapy and 22% injectable mediation; 81% preferred a therapy that can be taken at home.

**Conclusions:**

Despite current pharmacotherapies, patients reported substantial burden due to acromegaly and its treatment, which extends beyond clinical manifestations to impact activities, productivity, and HCRU.

## Introduction

Acromegaly is a chronic rare disease usually caused by a pituitary adenoma that secretes excessive amounts of growth hormone (GH), resulting in hypersecretion of insulin-like growth factor I (IGF-I) [1, 2]. Prolonged exposure to excess levels of IGF-I and GH can result in somatic disfigurement and a broad range of symptoms and comorbidities affecting almost all organ systems [1]. Acral enlargement and coarsening of facial features (enlargement of the nose and jaw, thickening of the lips, prominent brow ridge) are present in 65% to 90% of people with acromegaly and are highly suggestive of the diagnosis [1–4]. Other common signs and symptoms include tongue enlargement, headache, fatigue, hyperhidrosis, skin oiliness or thickening, and weight gain [1, 2, 4]. The majority of patients with acromegaly have multiple comorbidities, such as cardiovascular (e.g., cardiomyopathy, biventricular hypertrophy, hypertension), musculoskeletal (e.g., arthropathy, arthralgia, carpel tunnel syndrome), metabolic (e.g., diabetes, dyslipidemia), respiratory (e.g., excessive snoring, sleep apnea), reproductive (e.g., menstrual disturbance, endometrial polyps, erectile dysfunction), and gastrointestinal (e.g., colonic polyps) complications or disorders [1, 2, 4, 5]. The symptoms and complications associated with uncontrolled acromegaly impair patients’ quality of life (QoL) and contribute to premature death and a substantial socioeconomic burden [5–11].

Treatment goals include normalizing IGF-I and GH concentrations, controlling tumor growth, preventing and relieving disease-related symptoms and complications, reducing excess mortality, and improving QoL [2, 12, 13]. A multimodal approach comprising surgery, medical treatment, and in some cases, radiation therapy, is often required to achieve these goals [12, 13]. The preferred initial treatment for acromegaly is surgical removal of the adenoma [2, 12, 13]. While surgery provides biochemical remission for approximately 40% to 60% of patients [14], there is a need for medical therapy in those who refuse or are ineligible for surgery or who have an inadequate response to surgery [12–14].

Currently available medical treatment options include injected depot somatostatin receptor ligands (SRLs; SST2 dominant agonists, octreotide and lanreotide; SST5 dominant agonist, pasireotide), short-acting (subcutaneous) octreotide, oral octreotide, the oral dopamine receptor agonist cabergoline, and the subcutaneously administered GH receptor antagonist pegvisomant [2, 13, 15]. Injectable formulations of octreotide (short-acting or depot) and lanreotide are recommended as first-line medical therapy [2, 12, 13], and switching to oral octreotide can be considered for patients who respond to and tolerate treatment with injectable octreotide or lanreotide [2, 14]. Pasireotide and pegvisomant are generally used as second-line therapy in patients with an inadequate response to octreotide or lanreotide [2, 12, 13], although pasireotide may be considered ahead of other SRLs in patients with sparsely granulated tumors [16]. Pegvisomant can also be used in combination with an SRL [12–14]. Cabergoline monotherapy is mainly limited to patients with mild disease; adjunctive use includes combination therapy with octreotide or lanreotide [2, 12, 13].

Understanding treatment patterns and the burden associated with acromegaly and its treatment may help identify unmet needs in the management of this disease. The objective of this study was to elucidate the current management of patients with acromegaly and evaluate the real-world experience of disease burden, treatment burden, and healthcare resource utilization (HCRU) from the patient’s perspective in this clinical population.

## Methods

### Study design and patients

A quantitative, web-based survey was conducted in patients 18 to 75 years of age in the US, Canada, or the United Kingdom who previously underwent transsphenoidal surgery (TSS) and at the time of enrollment were receiving medical therapy for acromegaly. Patients were recruited through patient advocacy groups in the US following standard processes. The study included a background survey of patient characteristics, followed by a daily symptom survey completed for 90 days (results to be reported separately), and a final survey of patient experience with disease burden, medical therapies, and HCRU over the preceding 3-month period.

The acromegaly symptom items in the daily survey were developed in collaboration with patients and patient advocacy groups, in alignment with the Acromegaly Symptom Diary (ASD) tool. The development, psychometric evaluation, and validation of the ASD tool have been described previously [17]. The final survey contained the symptoms identified by patients as clinically important during development of the daily symptom survey, as well as additional signs and symptoms or medication side effects (coarse and thickened skin, abdominal pain, decreased libido, diarrhea, and injection site pain). The final survey also included questions on the extent to which acromegaly and acromegaly treatment interfered with life (reported using a numerical rating scale from 0 [no interference at all] to 10 [very significant interference]); the extent to which acromegaly treatment was burdensome (reported using a numerical rating scale from 0 [not at all burdensome] to 10 [extremely burdensome]); assessment of the absence or presence of prespecified symptoms experienced in relation to acromegaly and its treatment; the importance of prespecified treatment goals (rated from 0 [not at all important] to 10 [extremely important]); the extent to which patients were satisfied with prespecified aspects of acromegaly treatment (rated from 0 [not at all satisfied] to 10 [extremely satisfied]); the Work Productivity and Impairment Questionnaire (WPAI) [18], a validated patient-reported outcome scale; the impact of acromegaly on daily activities (reported on a scale from 0 [no interference at all] to 10 [very significant interference]); HRCU (acromegaly-related office visits, emergency department visits, and overnight hospitalizations); and patient preferences for administration-related attributes of acromegaly treatment.

The data collected met regulatory criteria for exemption from institutional review board (IRB) review due to anonymization; however IRB approval was still obtained for the daily symptom and final surveys assessing patient experiences. Patients provided consent for analysis of their survey results and received remuneration to compensate for their time for survey completion.

### Statistical analysis

Based on medical therapies for acromegaly reported during the previous 3 months, patients were categorized as receiving SRL depot injection monotherapy, SRL depot injection plus other treatment(s), or no SRL depot injection. Other treatments included pegvisomant, cabergoline (an oral medication used off-label for acromegaly), and oral or subcutaneous octreotide. Data were summarized using descriptive statistics. For questions assessing the impact, level of interference, or degree of burdensomeness of acromegaly or its treatment, responses were categorized as low/minimally (0–3), moderate/moderately [4–7], and high/highly [8–10, 19]. Scores on the WPAI were expressed as percent of impairment and productivity loss, with higher scores indicating greater impairment.

## Results

### Patients

Sixty-six patients completed the background survey, of whom 58 completed the final survey (analysis population). The daily symptom survey was completed by 40 patients (results to be reported separately). Most patients (51.7%) in the analysis population were between the ages of 41 and 60 years; 72.4% were female, 86.2% were White, and 82.8% were from the US (Table 1). The most common comorbidities were hypertension, hyperlipidemia, arthritis, and acid reflux.

**Table 1:** Patient characteristics

Characteristic	Patients (*n* = 58)
Age, n (%)	
21–40 41–60 61–80	13 (22.4) 30 (51.7) 15 (25.9)
Gender, n (%)	
Female Male Nonbinary Prefer not to say	42 (72.4) 14 (24.1) 1 (1.7) 1 (1.7)
Race/ethnicity, n (%)	
White Hispanic or Latino Asian American Other Prefer not to say	50 (86.2) 4 (6.9) 1 (1.7) 2 (3.4) 1 (1.7)
Geographic region, n (%)	
United States Canada United Kingdom	48 (82.8) 6 (10.3) 4 (6.9)
Common comorbidities, n (%)*	
Hypertension Hyperlipidemia Arthritis Acid reflux Hypothyroidism Type 2 diabetes	24 (41.4) 24 (41.4) 23 (39.7) 23 (39.7) 19 (32.8) 16 (27.6)

*Reported by ≥ 25% of patients

## Treatment history

During the previous 3 months, 36 (62.1%) patients had received depot SRL injections (18 as monotherapy and 18 in combination with other medications), and 22 (37.9%) patients had not received a depot SRL (Table 2). Overall, 21 (36.2%) patients had received depot lanreotide, 13 (22.4%) depot octreotide, and 6 (10.3%) depot pasireotide, including 4 patients who switched from one injected SRL to another during the 3-month study period. Among the 22 patients who had not received a depot SRL, 16 reported receiving monotherapy (oral octreotide [*n* = 4], cabergoline [*n* = 2], or pegvisomant [*n* = 10]), 3 received other combination therapy, and 3 reported not receiving an acromegaly medication.

**Table 2:** Medical therapies for acromegaly during the previous 3 months

Treatment, *n* (%)	Patients (*n* = 58)
**Depot SRL monotherapy**	**18 (31.0)**
Lanreotide depot	9 (15.5)
Octreotide LAR	6 (10.3)
Pasireotide LAR	3 (5.2)
Depot SRL + other treatment*	**18 (31.0)**
Lanreotide depot + other(s)	10 (17.2)
Octreotide LAR + other(s)	3 (5.2)
Lanreotide depot + octreotide LAR ± other(s)^†^	2 (3.4)
Octreotide LAR + pasireotide LAR ± other(s)^‡^	2 (3.4)
Pasireotide LAR + other(s)	1 (1.7)
**Other monotherapy**	**16 (27.6)**
Pegvisomant	10 (17.2)
Oral octreotide	4 (6.9)
Cabergoline	2 (3.4)
**Other combination therapy**	**3 (5.2)**
Cabergoline + pegvisomant	1 (1.7)
Oral octreotide + pegvisomant	1 (1.7)
Subcutaneous octreotide + pegvisomant	1 (1.7)
**No prescription acromegaly medication**	**3 (5.2)**

*Other treatments included pegvisomant, cabergoline, oral octreotide, and subcutaneous octreotide

^†^Lanreotide depot and octreotide LAR prescribed sequentially (i.e., medication change)

^‡^Octreotide LAR and pasireotide LAR prescribed sequentially (i.e., medication change)

LAR, long-acting release; SRL, somatostatin receptor ligand

### Disease and treatment burden

#### Disease burden

**Fig. 1:**
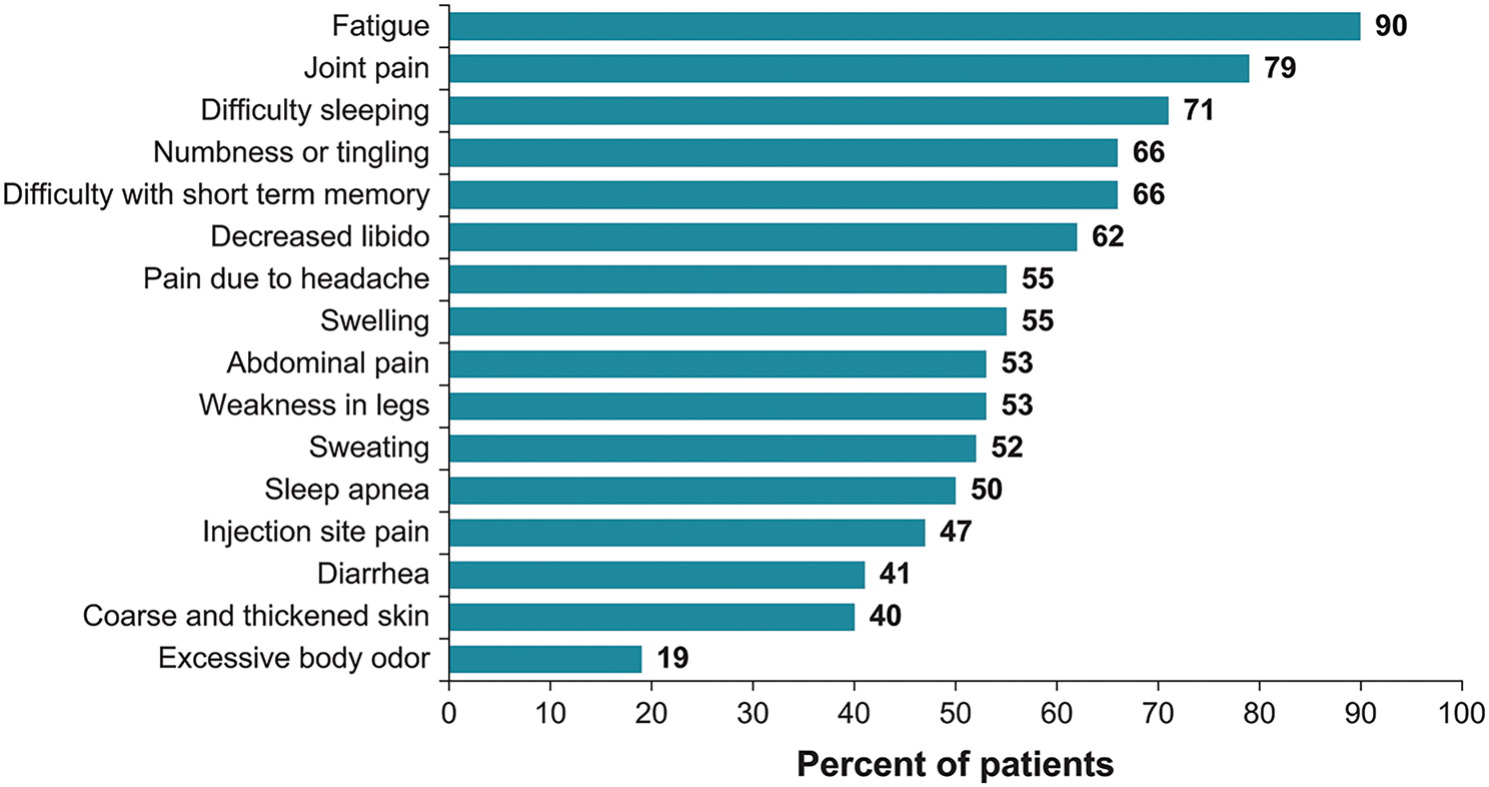
Symptom experience. Percentage of patients experiencing symptom(s) in response to the question: Over the last 3 months, which of the following symptoms have you experienced, if any?

In the final survey, patients were asked to recall their symptom experience over the prior 3 months. Despite treatment, most patients reported that acromegaly had a moderate (59%) or high (22%) level of interference in their life overall during the previous 3 months. Every patient recalled at least 1 symptom during the previous 3 months, and 67% recalled 1 or more symptoms with a severity rating of 8 or higher. The most commonly recalled symptoms were fatigue (90%) and joint pain (79%; Fig. 1). Symptoms recalled as highly severe by the greatest proportion of patients were joint pain (61%), fatigue (56%), decreased libido (50%), and leg weakness (42%). Among patients on SRL depot injection monotherapy (*n* = 18), 67% reported experiencing at least 1 breakthrough disease symptom at any time prior to the next injection, and 50% of these patients reported the breakthrough disease symptoms as highly burdensome. Of the 18 patients on combination therapy that included a depot SRL, 83% reported at least 1 breakthrough disease symptom, and 53% of these patients indicated that the breakthrough disease symptoms were highly burdensome. Most breakthrough symptoms were reported to occur at varying intervals throughout the treatment cycle.

**Fig. 2:**
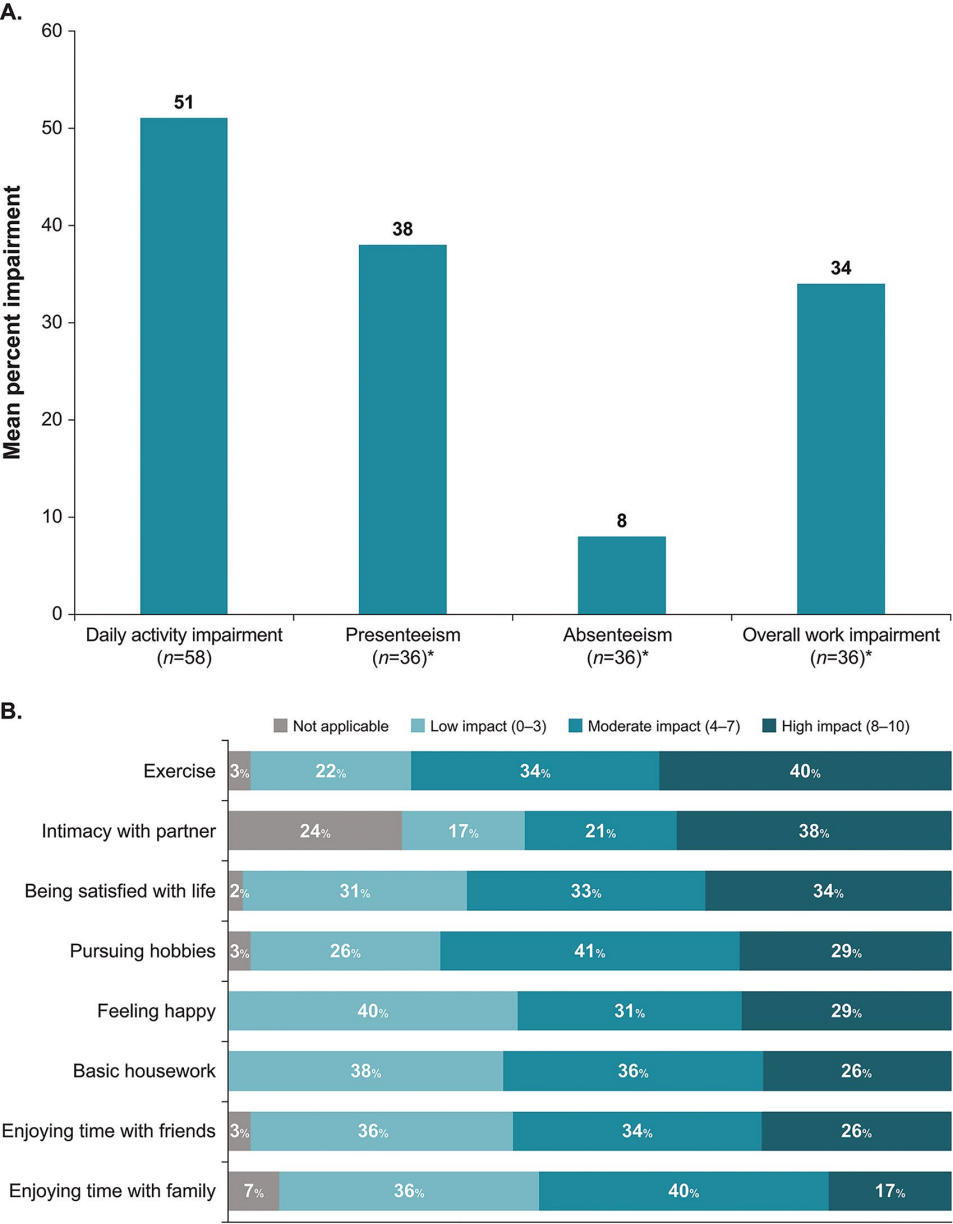
Impairments in functioning. (**A**) WPAI scores. Scores are expressed as percent of impairment and productivity loss, with higher scores indicating greater impairment. *Patients who were employed at the time of the survey. WPAI, Work Productivity and Activity Impairment questionnaire. (**B**) Interference of acromegaly with daily activities. Activities for which more than 50% of patients reported a moderate or high impact

As assessed using the WPAI (with recall over the past 7 days), acromegaly caused substantial impairments in daily activities and work productivity (Fig. 2A). A wide range of daily activities were negatively affected by acromegaly. Figure 2B presents activities for which more than 50% of patients reported a moderate or high impact.

Most patients (78%) reported receiving caregiver support for their acromegaly from sources that included a partner (60%), family member (57%), friend (31%), home health aide (3%), and support group (3%). Patients reported that caregivers provided support with day-to-day household activities (63%), rearranged their own schedules to provide support (48%), supported children/dependents under the patient’s care (41%), provided transportation to/from a healthcare center (35%), and administered patient’s injections (32%). As reported by patients, the time spent by caregivers was, on average, 9.6 h per week for household activities, 7.0 h per week supporting children/dependents, and 1.9 h per week providing healthcare-related transportation.

#### Treatment burden

**Fig. 3:**
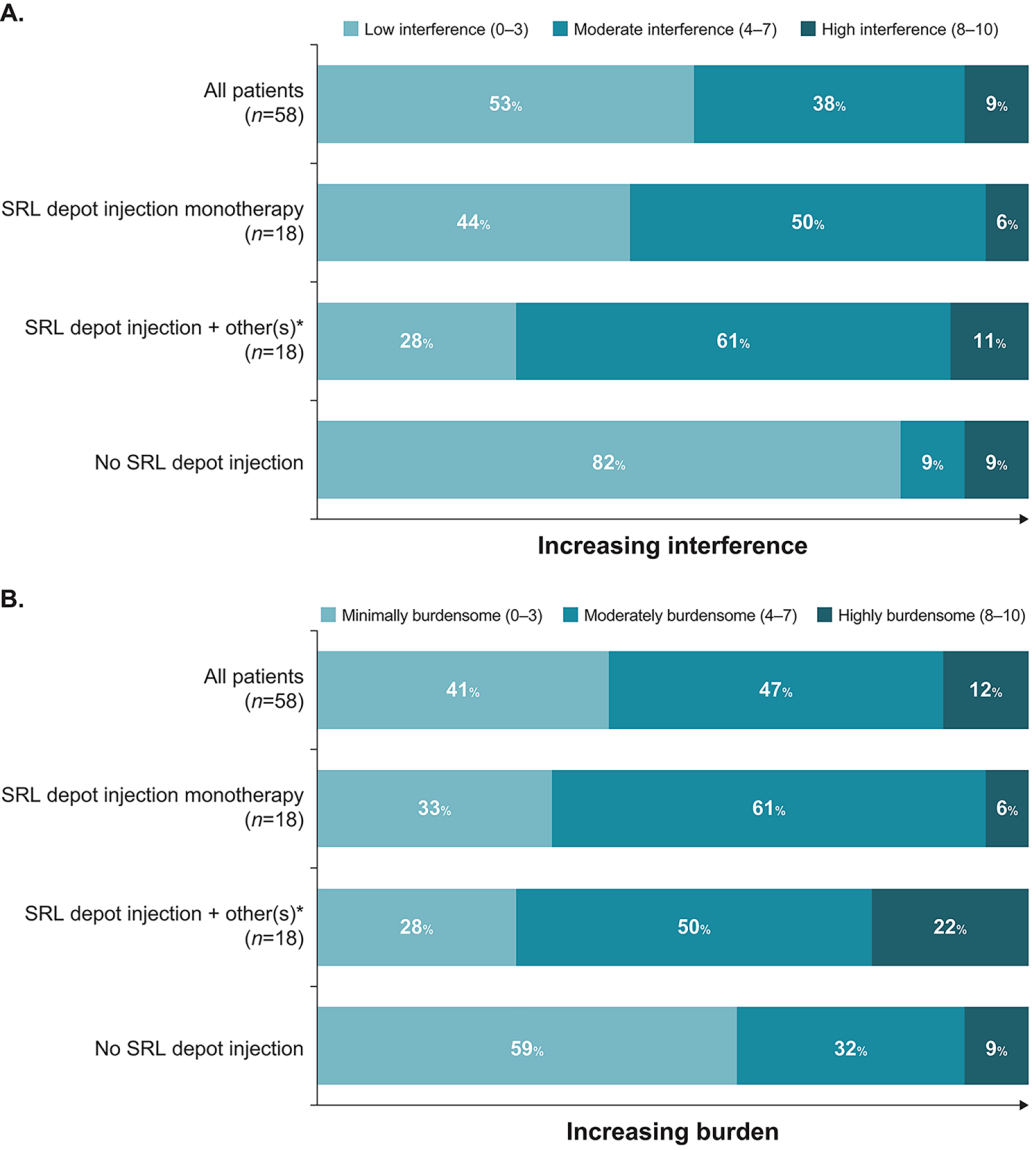
Burden of acromegaly treatment. (**A**) How much did acromegaly treatment interfere with life overall during the previous 3 months? (**B**) How burdensome is current acromegaly treatment? *Other treatments included pegvisomant, cabergoline, oral octreotide, and subcutaneous octreotide. SRL, somatostatin receptor ligand

Compared with patients not receiving depot SRLs, a higher proportion of those on SRL depot injections, whether as monotherapy or in combination with other treatments, reported that their acromegaly treatment moderately or greatly interfered with their life (56% and 72%, respectively, vs. 18%; Fig. 3A) and was moderately or highly burdensome (67% and 72%, respectively, vs. 41%; Fig. 3B).

### Healthcare resource utilization

During the previous 3 months, 83% of patients had at least 1 acromegaly-related outpatient visit (Table 3). The mean number of acromegaly-related outpatient visits was 2.6, with more frequent visits reported by patients receiving SRL depot injections. Among patients who had received SRL depot injections, octreotide LAR was administered by a healthcare provider at a hospital or clinic to 62% (8/13) of patients compared with 29% (6/21) of patients taking lanreotide LAR and 0% (0/6) of patients taking pasireotide LAR.

**Table 3:** Healthcare resource utilization during the previous 3 months

	All patients (*n* = 58)	SRL depot injection monotherapy (*n* = 18)	SRL depot injection + other(s)* (*n* = 18)	No SRL depot injection (*n* = 22)
Patients with ≥ 1 acromegaly-related office visit	48 (82.8)	16 (88.9)	17 (94.4)	15 (68.2)
Number of acromegaly-related office visits, mean	2.6	3.1	2.8	2.2
Patients with ≥ 1 emergency department visit related to acromegaly, n (%)	8 (13.8)	4 (22.2)	1 (5.6)	3 (13.6)
Patients with ≥ 1 overnight hospitalization related to acromegaly, n (%)	6 (10.3)	3 (16.7)	0 (0)	3 (13.6)

*Other treatments included pegvisomant, cabergoline, oral octreotide, and subcutaneous octreotide

SRL, somatostatin receptor ligand

### Treatment goals

**Fig. 4:**
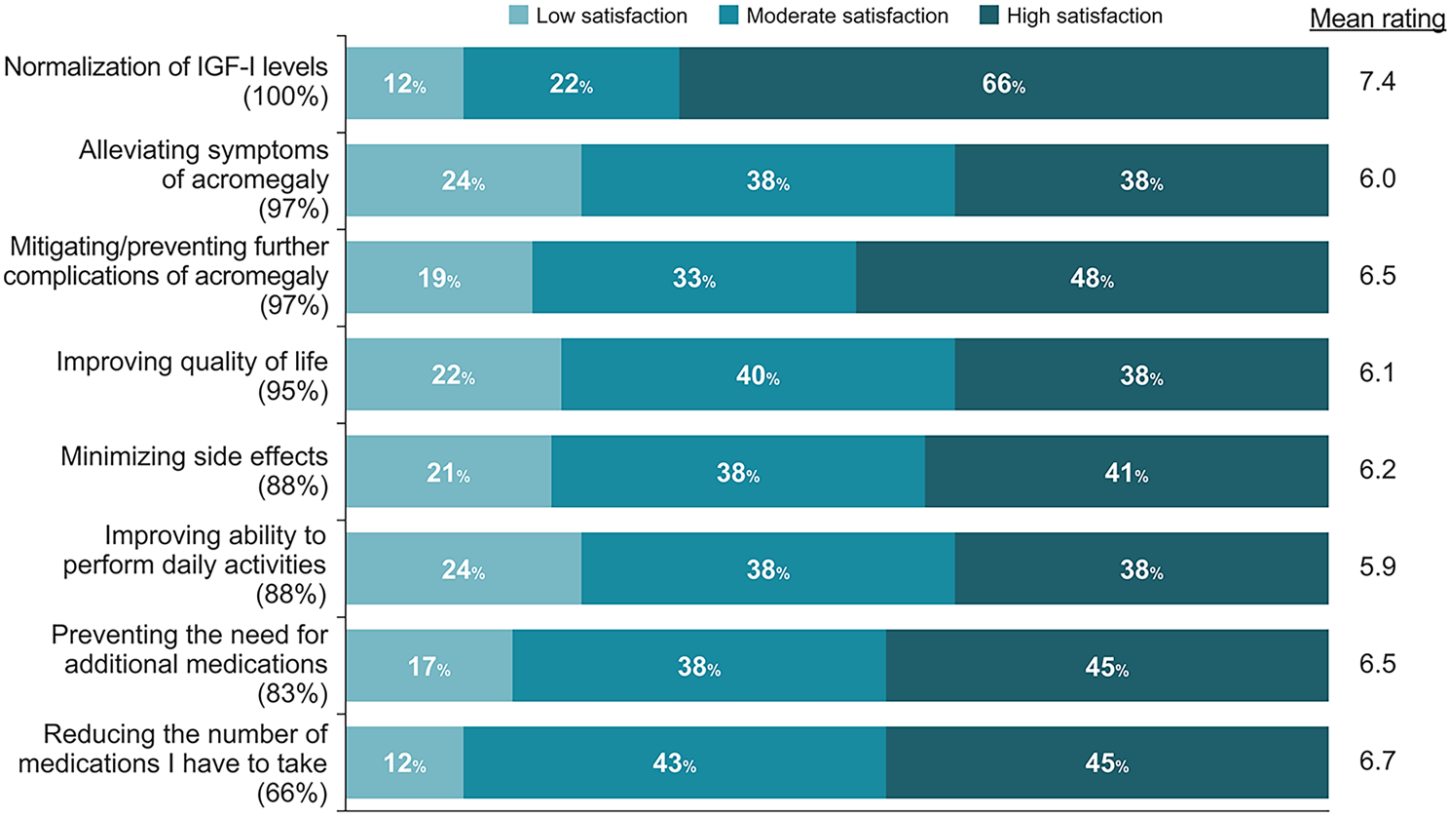
Satisfaction with treatment goals. Patients were asked: How important are the following when treating your acromegaly? Treatment goals are presented in descending order based on the percentage of patients who ranked the goal as being of “high importance” (percentage listed below each goal). Patients were then asked: How satisfied are you with the following aspects of your acromegaly treatment? IGF-I, insulin-like growth factor I

Patients identified normalization of IGF-I levels, alleviating symptoms of acromegaly, mitigating/preventing further complications of acromegaly, and improving QoL as the most important treatment goals (Fig. 4). Patients were most likely to report being satisfied with their treatment’s normalization of IGF-I levels, with 88% of patients reporting moderate-to-high levels of satisfaction (Fig. 4). Among the treatment goals identified as most important, alleviating symptoms of acromegaly and improving QoL were associated with moderate-to-high levels of satisfaction in 76% and 78% of patients, respectively.

### Treatment preferences

**Fig. 5:**
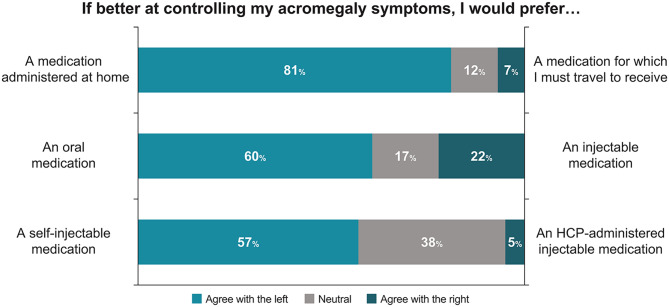
Patient preferences for acromegaly treatment. HCP, healthcare professional

For the treatment of acromegaly, patients expressed preference for an oral medication versus an injectable medication and for a medication that is administered at home rather than one that requires travel (Fig. 5). Overall, 71% of patients reported that they would be willing to consider new acromegaly treatments that could improve their symptoms.

## Discussion

This survey found that patients with acromegaly experienced a considerable disease burden over the previous 3 months despite treatment, with more than 80% of patients reporting a moderate or high degree of acromegaly interference in their life overall. All patients experienced symptom burden due to acromegaly. Fatigue and joint pain were consistently reported as the most common symptoms experienced, and two-thirds of patients reported at least 1 severe symptom that was not completely mitigated by treatment. Most patients receiving SRL depot injection monotherapy reported experiencing breakthrough disease symptoms, with half of patients considering these breakthrough symptoms to be highly burdensome. The interference and burden due to acromegaly extended beyond clinical manifestations to impact not only daily activities but also work productivity. Additionally, nearly 80% of patients reported receiving caregiver support to help manage their acromegaly-related needs, highlighting an under-recognized caregiver burden.

Current acromegaly treatments impose a substantial burden on patients. The majority of patients reported being treated with depot SRL injections, either as monotherapy or in combination with another treatment. Although these agents are generally recommended as first-line medical therapy [2, 12, 13], receiving depot SRLs was associated with a greater treatment burden, reflected by higher proportions of patients on depot SRLs reporting moderate-to-high interference of treatment on their lives and that their treatment was moderately to highly burdensome compared to patients whose treatment regimens did not include a depot SRL.

The findings of this survey suggest that, despite current standard of care (SOC), patients have symptoms that are not adequately managed, resulting in a high degree of impact and interference in their daily lives and functioning. More than half of patients reported a moderate to high impact of acromegaly on their ability to exercise, social functioning (intimacy with partner, enjoying time with friends and family), emotional well-being (being satisfied with life, feeling happy), and their ability to pursue hobbies and perform basic housework. Among employed patients, acromegaly was associated with considerable impacts on work productivity, with daily activity impairment (51%) and presenteeism (38%) being most strongly affected. Notably, the degree of overall work impairment among employed patients with acromegaly (34%) was higher than that observed in patients with chronic lung disease (25%), similar to that reported for cancer (31%) and cardiometabolic disease (37%), and slightly lower than that associated with pain (42%), migraine (44%), and depression (47%) [20, 21]. Patients identified normalizing IGF-I levels, alleviating symptoms of acromegaly, mitigating/preventing further complications, and improving QoL as the most important treatment goals.

Overall, more than 80% of patients had at least one acromegaly-related doctor’s visit and visited the doctor 2.6 times on average in the prior 3 months, with greater acromegaly-related outpatient usage observed among those patients receiving depot SRLs. The financial implications of both the direct costs of HCRU and indirect costs of reduction in work productivity reveals an economic burden on patients, caregivers, payers, and the healthcare system that is not well documented in the current scientific literature on acromegaly therapy burden.

Although the sample size was small, the findings of the current survey align with previously reported observations regarding the treatment history and disease burden of acromegaly in patients with the disease, providing evidence of the unmet need in this patient population. A retrospective analysis of data from a large US commercial claims database (MarketScan) reported broad use of injectable depot SRLs through 3 lines of treatment, although cabergoline was commonly used in the first-line setting [22]. Surveys of patients with acromegaly receiving SRL depot injections have reported high rates of acromegaly symptoms despite treatment, with the most frequently reported symptoms being joint pain, soft tissue swelling, fatigue/tiredness, headache, snoring, and excessive sweating [23, 24]. In one study, 87% of patients reported that they experienced symptoms that interfered with daily activities despite treatment with injectable depot SRLs [23]. Similarly, a focus group composed of 7 patients with acromegaly found that patients reported a limited capacity for daily activities and responsibilities (e.g., laundry, grocery shopping), due in part to impaired mobility and difficulties with manual agility and sensitivity [25]. Patients also reported that tiredness/fatigue limited their ability to engage in work and leisure activities.

The presence and burden of residual acromegaly symptoms in the current survey and in previous studies [23, 24] highlight the problem of therapeutic inertia in the management of acromegaly. In large-scale surveys, only about half of patients achieved biochemical control at 1 year after diagnosis or pituitary surgery, and approximately 45% to 80% of patients with acromegaly had long-term biochemical control [26–28]. Factors such as delays in identifying therapy-resistant cases, failure to properly titrate drugs and individualize therapy, and settling for partial disease control may contribute to therapeutic inertia. In an analysis of data from the screening phase of a phase 3, randomized trial of oral octreotide capsules (MPOWERED), patients reported acromegaly symptoms that interfered with their daily activities despite being on a stable dose of depot SRLs, with significantly lower interference among patients with both IGF-I and GH levels within the normal range compared with patients with elevated IGF-I or GH levels. These findings underscore the importance of achieving and maintaining complete biochemical control in order to minimize the disease burden of acromegaly [14].

Currently available medical therapies for acromegaly are associated with several important limitations. Depot injections commonly produce injection-site pain that may last for hours or days (perhaps even weeks after intramuscular injections), as well as other injection-site reactions, such as bruising, swelling, nodules, and scar tissue at the injection site [14, 23, 24]. Administration of depot injections by healthcare professionals may necessitate repeat office visits [14, 23]. Additionally, depot injections may result in inaccurate drug delivery [29] and variable biochemical and symptom control throughout the dosing interval [24, 30]. In the current study, two-thirds of patients receiving depot SRLs reported breakthrough symptoms prior to their next injection, and half of these patients indicated that the breakthrough symptoms were highly burdensome. These observations are consistent with those of previous studies, in which breakthrough symptoms have been reported toward the end of the treatment cycle [7, 23–25]. Pasireotide is also limited by its propensity to cause or exacerbate hyperglycemia and type 2 diabetes [12, 31]. Cabergoline is used off-label for acromegaly but, due to modest efficacy, its use is often limited to patients with mild disease or as an adjunct to SRL therapy. Administration requirements of non-depot medications may also be burdensome. Oral octreotide is administered twice daily, with a fasting period required for each dose [32]. Pegvisomant requires daily subcutaneous injections, which may be burdensome for some patients [33].

Patients in the current study expressed a preference for an oral medication versus an injectable medication and for a medication that can be administered at home. Similarly, 48% of patients in an international survey (*N* = 195) who received depot SRLs and 85% of patients in a US-based survey (*N* = 106) receiving various acromegaly medications indicated a preference for oral therapy [7, 24]. In a survey of 109 US adults with acromegaly, patients reported that they would prefer subcutaneous injections administered with a pen at home once every 4 weeks over twice-daily oral administration requiring fasting before and after each dose (i.e., as with oral octreotide) [34].

While all patients in this study had undergone TSS and were receiving medical therapy, it is important to consider healthcare access challenges and social determinants of health that may contribute to the burden of disease in diverse patient populations. Lack of access to experienced neurosurgeons without a long waitlist is a barrier to undergoing TSS in a timely manner. A systematic review of US studies of patients with pituitary adenomas identified racial and socioeconomic status [35]. Further, access to high-quality education and care, including multidisciplinary management of acromegaly, may differ among patient populations and may potentially influence outcomes [36].

Despite the availability of several standard-of-care medical therapies, many patients remain untreated or undertreated, contributing to the high socioeconomic burden of acromegaly. A retrospective, cross-sectional administrative claims analysis of a US representative database (IQVIA Pharmetrics Plus^®^) that followed adult patients (with newly diagnosed or pre-existing acromegaly) for 1 year after a randomly selected claim with an acromegaly diagnosis found that most patients (59%) lacked evidence of therapy (pituitary surgery, radiation treatment, or medical treatment) during the follow-up period [37]. Among patients with acromegaly therapy, the most common treatments were SRLs (48.9%) and dopamine receptor agonists (35.3%). Mean total healthcare costs and hospitalization rates were both 4-fold higher among patients with acromegaly versus the population without acromegaly, indicating a high burden of disease. These findings suggest disparities in access to effective treatment options following diagnosis and highlight the importance of identifying and addressing barriers to treatment choice in order to improve patient outcomes.

The current study had several key limitations. As is inherent to real-world studies, patients were not randomly assigned to treatment, and it is possible that patient factors such as disease severity, comorbidities, and history of response to treatment affected both the current treatment regimen and the assessed outcomes. Acromegaly is a rare disease, so the small sample size (*n* = 58) precluded meaningful comparison of baseline demographics and clinical characteristics across treatment groups and may also limit generalizability of the study findings. Further, the preponderance of US respondents (82.8%) and White participants (86.2%) may have biased the results. Although a higher incidence of pituitary adenomas has been reported among Black populations compared with White or other racial groups, racial disparities have been described, with Black patients less likely to undergo TSS [38]. This may have resulted in underrepresentation of Black patients in the current study, since having previous TSS was a participation requirement. Patients in the current study were recruited through patient advocacy groups in the US; future studies that recruit patients internationally through advocacy groups and/or medical practices in different countries may achieve a more geographically and racially diverse patient sample. Additionally, details of patients’ clinical and medication histories, other than those reported herein, were unavailable.

In the final survey, patients were asked to recall symptoms over a 3-month period, which may have introduced recall bias [39], especially given that acromegaly is a chronic condition with fluctuating symptoms [40]. As described above, patients in this study also completed a daily symptom survey for 90 days, and results of this survey will be reported separately and compared with findings from the current analysis. Objective clinical data, such as biochemical control, were not available. The absence of IGF-I and GH data limits the ability to evaluate any correlation between disease burden and biochemical disease control. Previous studies examining the relationship between biochemical control and symptom burden have yielded conflicting results [14, 23, 24, 41–45]. For example, many patients treated with depot SRL injections report the occurrence of breakthrough acromegaly symptoms even when IGF-I is normal [23, 24]. More research integrating objective clinical outcomes with patient-reported data is warranted. Finally, as medication nonadherence is often problematic in patients with acromegaly [8, 22], it is possible that the residual disease burden in some patients was due to nonadherence with prescribed medication.

Beyond examining biochemical control and other laboratory assessments, there is a need to better understand patients’ symptoms and the frequency with which acromegaly symptoms and treatment impact and interfere with patients’ lives. This study, performed in a real-world population of patients with acromegaly, aimed to understand the patient experience of the burden of acromegaly, including symptoms, and its treatment from the patient’s perspective. Strengths of the study include assessment across a wide range of patient experiences (i.e., symptoms, treatment experience, HCRU, and impact on the ability to work) and participation by patients taking various medication regimens, which allowed comparisons between regimen types.

Several products are in development that may potentially reduce the clinical burden of acromegaly and its treatment. Paltusotine (Crinetics Pharmaceuticals; San Diego, CA) is currently under US Food and Drug Administration (FDA) review for the treatment of acromegaly and, if approved, will be the first once-daily, oral, nonpeptide, selective somatostatin receptor type 2 agonist available for this indication [46–49]. Phase 3 clinical trials of paltusotine included a broad patient population with acromegaly encountered in clinical practice, including patients biochemically controlled on injectable SRLs (PATHFNDR-1) [50] and patients who are pharmacologically untreated and biochemically uncontrolled (PATHFNDR-2) [51]. Once-daily oral paltusotine demonstrated superior maintenance of biochemical control (IGF-I ≤ upper limit of normal [ULN]: 83.3% vs. 3.6%, *p* < 0.0001) and symptom control versus placebo in patients who switched from an injected SRL [50], achieved rapid and sustained biochemical and symptom improvement compared with placebo in previously untreated or treated/washed out patients with biochemically uncontrolled acromegaly [51], and was well tolerated. The rapid onset of paltusotine’s effects may allow for timely assessment of biochemical effectiveness relative to long-acting injectable formulations, while its flexible dosing, which enables quick upward or downward titration, may support more personalized treatment management [51].

A novel formulation of injectable (depot) subcutaneous octreotide (CAM2029; Camurus, Princeton, NJ) was developed using FluidCrystal technology [52]. A phase 3 clinical trial (ACROINNOVA 1) in 72 patients with acromegaly who had previously demonstrated biochemical control on depot SRL monotherapy found superior biochemical control with CAM2029 compared with placebo (IGF-I ≤ ULN; 72.2% vs. 37.5%, *p* = 0.0018) and a safety profile consistent with SOC [52]. Patients treated with CAM2029 reported improvements from baseline (during SOC treatment) in QoL and treatment satisfaction. Notably, CAM2029 has not been evaluated in treatment-naïve or treated/washed out patients with biochemically uncontrolled acromegaly.

Pegvisomant, the only currently available GH receptor antagonist, is administered as a daily subcutaneous injection [33]. Two additional GH receptor antagonists are under development, including site 1−binding helix (S1H, also an antagonist of prolactin receptors; in preclinical development) [53, 54] and ALXN2420 (formerly AZP-3813; Alexion Pharmaceuticals, Boston, MA; in phase 1 clinical development as add-on therapy in patients inadequately controlled with SRLs) [55–57]. In addition, a phase 1 clinical trial of MAR002 (an anti-GH receptor antibody; Marea Therapeutics, San Francisco, CA) was initiated in August 2025.

## Conclusions

Despite receiving currently available medical therapies for acromegaly, survey respondents reported substantial burdens due to acromegaly and its treatment. These burdens extended beyond clinical manifestations to include negative effects on daily activities and work productivity. Utilization of SRL depot injections was associated with a greater treatment-related burden and more office visits, and the majority of respondents expressed preference for an oral medication. A number of products are in development that may help to lessen the clinical burden of acromegaly and its treatment. Further research on treatment patterns, switching, and costs of care associated with uncontrolled acromegaly is underway.

## Data Availability

The datasets used and/or analyzed during the current study are available from the corresponding author on reasonable request.

## Abbreviations

ASD: Acromegaly Symptom Diary
FDA: US Food and Drug Administration
GH: Growth hormone
HCRU: Healthcare resource utilization
IGF-I: Insulin-like growth factor I
IRB: Institutional review board
QoL: Quality of life
LAR: Long-acting release
S1H: Site 1−binding helix
SOC: Standard of care
SRL: Somatostatin receptor ligand
SST: Somatostatin receptor
TSS: Transsphenoidal surgery
ULN: Upper limit of normal
WPAI: Work Productivity and Impairment Questionnaire
